# Temperate insects with narrow seasonal activity periods can be as vulnerable to climate change as tropical insect  species

**DOI:** 10.1038/s41598-020-65608-7

**Published:** 2020-06-01

**Authors:** Frank Johansson, Germán Orizaola, Viktor Nilsson-Örtman

**Affiliations:** 1 Uppsala University, Animal Ecology, Department of Ecology and Genetics, Evolutionary Biology Center, Norbyvägen 18D, S-75236 Uppsala, Sweden; 2 IMIB-Biodiversity Research Institute (Univ. Oviedo-CSIC-Princ. Asturias), c/ Gonzalo Gutiérrez Quirós s/n, 33600 Mieres-Asturias, Spain; 3 University of Oviedo, Zoology Unit, Dept Biology of Organisms and Systems, c/Rodrigo Uría s/n, 33071 Oviedo-Asturias, Spain; 4 Lund University, Department of Biology, Evolutionary Ecology Unit, Sölvegatan 12, S-22362 Lund, Sweden

**Keywords:** Evolution, Climate change, Climate-change impacts

## Abstract

The magnitude and ecological impact of climate change varies with latitude. Several recent models have shown that tropical ectotherms face the greatest risk from warming because they currently experience temperatures much closer to their physiological optimum than temperate taxa. Even a small increase in temperature may thus result in steep fitness declines in tropical species but increased fitness in temperate species. This prediction, however, is based on a model that does not account for latitudinal differences in activity periods. Temperate species in particular may often experience considerably higher temperatures than expected during the active season. Here, we integrate data on insect warming tolerance and temperature-dependent development to re-evaluate latitudinal trends in thermal safety margins after accounting for latitudinal trends in insect seasonal activity. Our analyses suggest that temperate and tropical species differ far less in thermal safety margins than commonly assumed, and add to the recent number of studies suggesting that tropical and temperate species might face similar levels of threat from climate change.

## Introduction

Climate change is expected to have a severe impact on the earth’s biodiversity and ecosystems^1^. In fact, numerous studies have documented pronounced shifts in the phenology, physiology and distribution of plant and animal species associated with recent changes in climatic conditions^2–7^. As a consequence, there is an urgent need to develop methods that can predict the impact of future climate change on populations and species at a global scale. Ideally, these methods should be able to identify broad geographic or taxonomic trends in the susceptibility of organisms to climate change, thereby helping to guide efforts to alleviate the consequences of climate change warming more effectively^3,8^. While considerable progress has been made toward developing such a framework^9–12^, many challenges remain.

**Figure 1:**
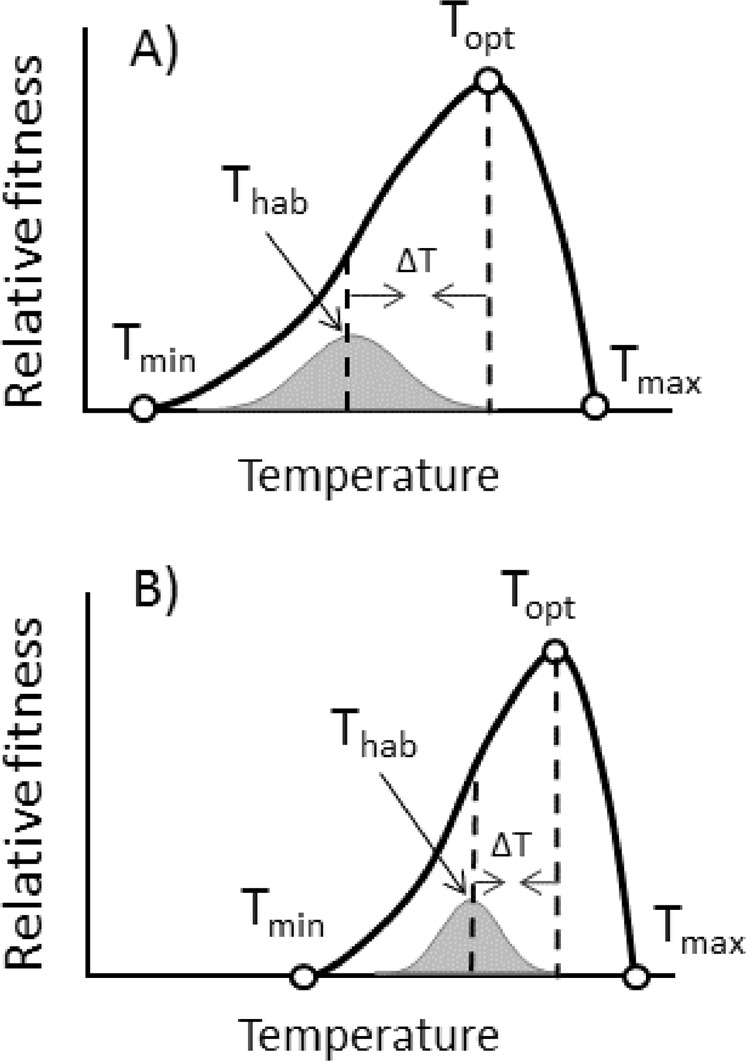
Thermal performance curves of ectotherms, here depicted as relative fitness. (**A,B**) represent a temperate and a tropical species, respectively. T_min_ and T_max_ represent the minimum and maximum temperature at which organisms can perform, and T_opt_ is the optimal temperature for performance. The grey curve beneath the thermal performance curve is the temperature that the organism is exposed to during an average year and the average is depicted as T_hab_. ΔT is the distance between T_hab_ and T_opt_. With climate change this distance decreases and might even be shifted to the right of T_opt_ or T_max_^11^. Tropical species are assumed to be temperature specialists (**B**), because they have narrower thermal performance curves and, therefore, are predicted to be more sensitive to climate change^11^.

In this emerging framework, thermal performance curves (TPCs) have become a key component (Fig. 1). TPCs describe how temperature affects an organisms’ fitness or key contributing functions, such as locomotion, growth and reproduction^12,13^. TPCs are especially relevant for ectotherms – the most diverse and widespread group of terrestrial animals – because ambient temperature has a direct and profound impact on nearly all aspects of ectotherm performance. TPCs for ectotherm performance and fitness typically show a gradual increase in performance from a critical thermal minimum (T_min_) where performance or fitness is zero, until reaching an optimal temperature (T_opt_) where performance is highest, before decreasing rapidly towards a critical thermal maximum (T_max_) (Fig. 1).

In the last decade several studies have synthesized and analyzed ectotherm TPC data on a global scale. Most strikingly, these studies have brought widespread attention to the fact that tropical species appear to be more vulnerable to climate change than temperate species. For example, in a seminal paper, Deutsch *et al*.^11^ used TPCs for insect fitness to estimate two measures of an organism’s susceptibility to warming: the amount of warming that an organism can tolerate before it experiences a decline in fitness (thermal safety margin: TSM) or reach zero fitness (warming tolerance: WT). The results revealed that tropical species have considerably narrower thermal safety margins and warming tolerances than temperate species^11^. This suggests that even a small increase in global mean temperatures will result in precipitous declines in the fitness and performance of tropical species – as they become pushed toward or beyond their thermal maxima – whereas temperate species will benefit from an increase in mean temperatures, as they currently experience temperatures well below their thermal optimum^14–16^). Later studies, employing a similar methodology, have broadly supported these conclusions^4,17–21^.

However, more recent studies have found that mid latitude species might be the most vulnerable^21–25^. Using a thermal safety margin index Kingsolver *et al*.^21^ predicted that a great proportion of mid-latitude insect species would experience declines in fitness due to climate change. Similarly, Pinsky *et al*.^25^ showed that mid latitude terrestrial ectotherms, had the lowest thermal safety margin across a latitudinal gradient covering temperate and tropical species. In contrast, and in support of past studies, they found that the marine ectotherms at the equator had the lowest thermal safety margins^25^. Focusing on intraspecific plastic and evolved differences Diamond *et al*.^23^ concluded that the typical biogeographic pattern of high vulnerability in the tropics is exacerbated for some sources of variation while for other sources of variation, including certain types of plastic variation in heat tolerance, the biogeographic pattern of high tropical vulnerability was weak. Before a consensus can be drawn whether tropical or temperature species are more sensitive to climate change, more studies are needed. We provide one such study by reanalyzing the data set used by Deutsch *et al*.^11^. One strength of this data set is that it uses intrinsic rates of population growth (r), a direct measure of Darwinian fitness.

The prediction made by Deutsch *et al*.^11^ that tropical species are more vulnerable to warming than temperate taxa, rests on several important assumptions. Here, we examine one of these assumptions, namely that the duration of the active period of insects is similar across latitudes. This assumption arises from using annual climatological data when performing these analyses. In other words, in many studies, estimates of T_opt_ and T_max_ are compared with the mean habitat temperature across the year at each location. However, because most ectotherms become inactive during the parts of the year when conditions are unfavorable^26^, most organisms will tend to experience warmer and less variable temperatures during the active season than expected based on annual means and variances. Deutsch *et al*.^11^ were aware of this, and complemented their original analyses (based on annual mean temperatures) with an analysis were they calculated WTs and TSMs using the mean temperature during the three warmest months of the year at each location. Overall, this did not change their conclusions regarding latitudinal trends in the susceptibility to warming. However, in these complementary analyses, temperate and tropical organisms were assumed to have identical seasonal activity patterns (and thus become inactive during a major part of the year). This contrasts with the empirical evidence, which shows clear latitudinal trends in ectotherm activity patterns^26,27^. In particular, temperate insects display a much stronger association between insect activity and temperature, meaning that the mismatch between the annual climate and that experienced by individuals during the active season is likely to be greatest for insects from higher latitudes. Whether restricting the analysis to the three warmest months of the year (as in^11^ or the six warmest months (as in^21^ re-analysis of the Deutsch *et al*. dataset) represents a realistic approximation of the thermal environment experienced by active insects across latitude remains unknown. Taking the biologically active period of insects and other ectotherms into consideration could thus be critical for generating more precise and biologically relevant predictions for the effects of climate change across latitudes. However, to our knowledge, no study has assessed the consequences of empirically-observed latitudinal differences in activity periods on the vulnerability to warming at a global scale.

Here, we re-examine the prediction that tropical species currently experience mean habitat temperatures that are much closer to their thermal optimum and maximum than temperate species and thus are at a greater risk from warming. To do this, we revisit the dataset used by Deutsch *et al*.^11^ and test for differences between tropical and temperate species in the vulnerability to climate change, after accounting for empirically-based estimates of insect active periods across latitudes. Admittedly, our analyses – as well as those of Deutsch *et al*.^11^ – ignore several additional factors that are increasingly known to be important for ectothermic responses to climate change, including thermoregulatory behavior^28^, capacity for thermal acclimation^29^, non-linear effects of temperature variance^30^, response to the duration and intensity of extreme temperatures^31^, shifts in phenology^27,32^, genetic (co)variances^33^ and interspecific interactions^34^. Nevertheless, by focusing specifically on the assumption that active periods are similar across latitudes, we explore the importance of accounting for insect activity periods when deriving predictions for the vulnerability of populations and species across latitude to future climate change.

**Figure 2:**
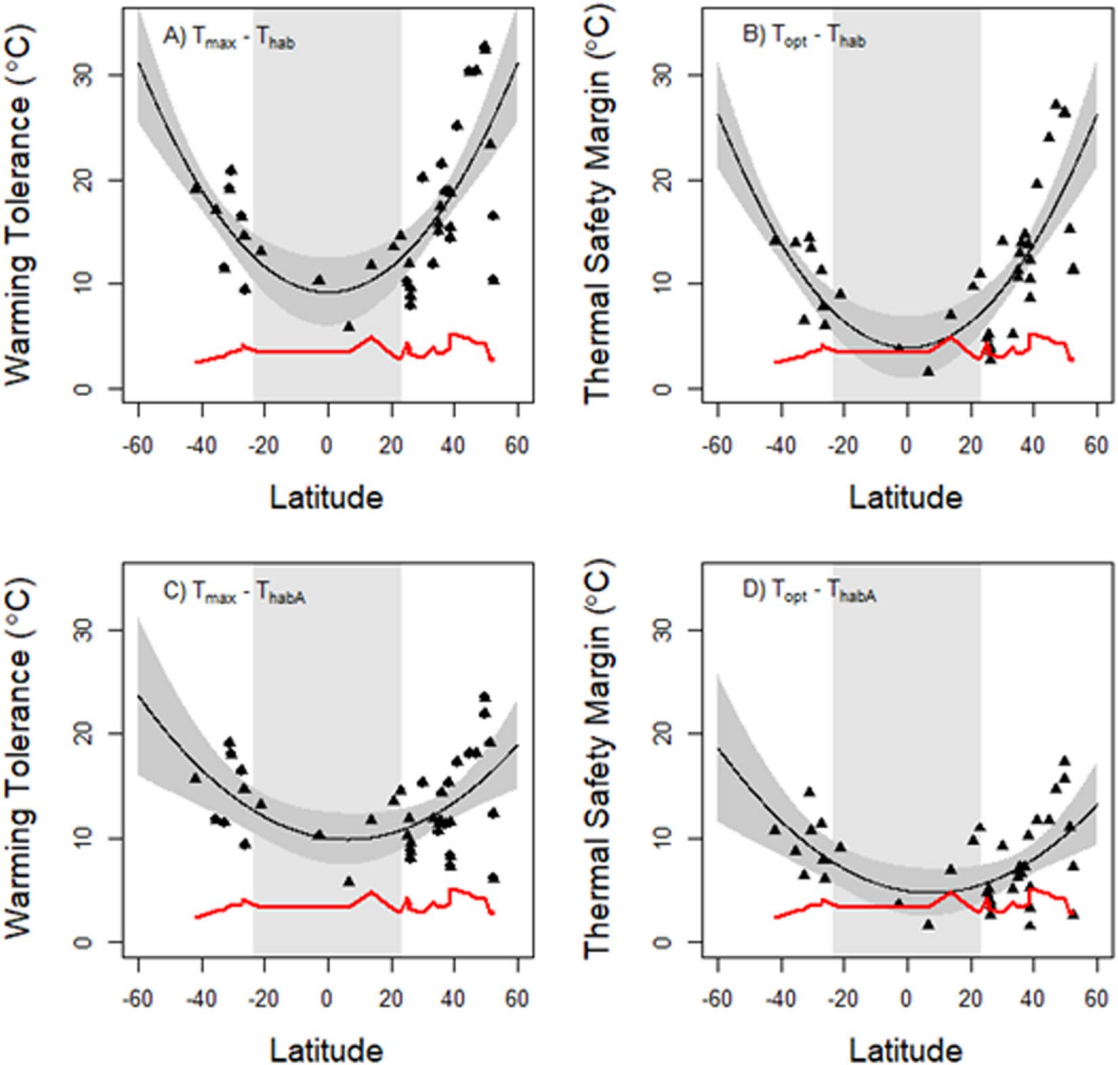
Warming tolerances (WT) and thermal safety margins (TSM) of insects as a function of latitude. In (**A,B)** warming tolerances and thermal safety margins were calculated using the mean annual temperature T_hab_ as in several previous studies (i.e. WT = T_max_ − T_hab;_ TSM = T_opt_ − T_hab_). In (**C,D**), warming tolerances and thermal safety margins were calculated using the mean temperature during the months of the year when insects are developmentally active, T_habA_ (i.e. WT = T_max_ − T_habA;_ TSM = T_opt_ − T_habA_). The red line represents the predicted increase in mean temperature at each latitude by 2080. Species whose data points fall below this line in (**B,D**) are predicted to experience mean habitat temperature that exceed their T_opt_ in 2080, and thus decline in fitness. Note that species whose data points fall near the red line may also experience fitness declines under climate change due to thermal fluctuations that exceed T_opt_. Tropical areas (defined here as regions located between 23°N and 23°S latitude) are shaded in light gray. Predictions from a second-degree polynomial model for the latitudinal trend in WT and TSM are shown in black; 95% confidence intervals for the predictions are shown in dark gray.

We define the active period of each studied insect population as the months of the year at a given location when the average temperature falls above the lower thermal threshold for insect development. After establishing the active period at each location, we re-calculate warming tolerances (WT) and thermal safety margins (TSM) using both the annual mean habitat temperature (T_hab_) originally used by Deutsch *et al*.^11^ and a new metric described here; the annual mean habitat temperature during the active period T_habA._ When we account for differences in insect activity periods across latitudes, we predict that: 1) temperate species will experience temperatures much closer to their T_opt_ and T_max_ than previously assumed (Fig. 1); 2) temperate and tropical species will thus have more similar warming tolerances and thermal safety margins than expected based on the mean annual temperature; and 3) temperate and tropical species will face a similar risk of experiencing fitness declines under future climate change scenarios.

## Material and methods

We combined two global eco-physiological datasets to re-evaluate the impact of climate change on insects across latitudes: the dataset on insect thermal tolerance previously analyzed by Deutsch *et al*.^11^ and a dataset on temperature thresholds of insect development published by Dixon *et al*.^35^. Importantly for this study, the Deutsch *et al*.^11^ dataset contain estimates of T_opt_ and T_max_ derived from TPCs for insect fitness (intrinsic population growth rate) from 38 insect species globally, and the Dixon *et al*.^35^ dataset contain estimates of the minimum developmental temperature for 66 species of insects. Briefly, our analysis followed a four-step procedure. First, we defined the lower thermal threshold for insect development (T_dmin_) using data from Dixon *et al*.^35^. Second, we defined the active period of each insect population in the Deutsch *et al*.^11^ dataset as the months of the year when the mean habitat temperature fell above T_dmin_. Third, we calculated a novel metric, T_habA,_ which we defined as the mean habitat temperature during the active period for each population in the Deutsch *et al*.^11^ dataset. Finally, we re-calculated the warming tolerance WT as T_max_ − T_habA_ and the thermal safety margin TSM as T_opt_ − T_habA_ for each population and plotted these metrics against latitude. We describe the analysis in greater detail next.

### Minimum developmental temperature (T_dmin_)

Insects cannot develop below a certain temperature^35^. Therefore, the majority of insects at high latitudes are only active during the warmer months in a year and enter diapause at some point near the end of the growth season^26,36^. However, the start and end of the active season is usually not determined by temperature directly. Instead, diapause is most often triggered by photoperiodic cues, as photoperiod tends to be a much more reliable cue of long-term mean climatic conditions – and hence expected future conditions – than the current temperature alone^26,36^. We therefore expect photoperiodic responses to have evolved so that organisms enter diapause at the time of the year when the long-term mean temperature falls at, or slightly above, the lower developmental threshold.

Based on this, we used the months when the mean temperature lies above the lower developmental threshold temperature for insects (T_dmin_) as a biologically plausible estimate of the active period of a population. Doing so ensures that shorter periods of cold temperatures that occur within the active period will count toward the thermal conditions experienced by active insects, whereas warmer periods that occur outside these months will not (because an individual will then be in diapause).

In a review, including 66 species in 8 insect orders, Dixon *et al*.^35^ reported a mean T_dmin_ of 13.3 °C across all insects. Analyzing data from the original studies, we explored whether T_dmin_ changed systematically with latitude. We were able to retrieve data on the latitude of collection for 36 of the species in Dixon *et al*.^35^ (Supporting information, Table S1). A regression analysis of these 36 species showed no significant relationship between T_dmin_ and absolute latitude (r^2^ = 0.07, P = 0.12). However, there was a tendency for high latitude species to have somewhat lower T_dmin_ (slope =  − 0.08) than low latitude species. Mean and median T_dmin_ for species collected above or below 40° N in this data set was 8.9 and 11.4, respectively. Using the mean T_dmin_ from the original data in Dixon *et al*.^35^, i.e. 13.3 °C, could thus introduce a bias in that northern species would appear to be relatively more sensitive to warming. We therefore use the mid-range value between the mean and the median, 10 °C, as a conservative estimate of T_dmin_ for all species when calculating the mean ambient temperature experienced during the active period. An alternative for the 10 °C limit would be to use the actual threshold for all the 38 species we use, but unfortunately this would drop our replicate number considerable. Our mean ambient estimate is approximately the same as that used in recent studies on effects of climate change on insects, e.g. Buckley *et al*.^27^, and the number of dropped months compared to Deutsch *et al*.^11^ can be seen in Supporting information, Table S2.

### Habitat temperature during the active period (T_habA_)

Insects are ectotherms and therefore the body temperature of most species closely matches that of their habitat in the absence of behavioral thermoregulation^37^. Based on this, we calculated T_habA_ as the mean air temperature of those months were the temperature was above 10 °C for the 38 species of insects that were provided in Deutsch *et al*.^11^. We were unable to retrieve the exact same observational climatic dataset as that used by Deutsch *et al*.^11^. We therefore used a slightly different set of observational climatic data (the coordinates for the climate data set differ slightly at the collection location). To determine if our climatic dataset was comparable to that used by Deutsch *et al*.^11^, we re-calculated T_hab_ using our climatic dataset and compared the results with those originally presented by Deutsch *et al*.^11^. The results were highly congruent (Supporting information, Table S2).

### Climate data

Monthly baseline climate data for the 20^th^ century (1901–2009) for each month was obtained from (https://climateknowledgeportal.worldbank.org). The data comes from the globally available observational datasets derived from the Climate Research Unit (CRU) of the University of East Anglia (http://www.cru.uea.ac.uk/data). For the future global warming we used the predicted mean monthly temperate for the year 2080 using, as in Deutsch *et al*.^11^, the simulation from the Geophysical Fluid Dynamics Laboratory model CM2.1^38^ that is forced by the A2 greenhouse gas emissions scenario (obtained at https://climatewizard.ciat.cgiar.org) based on grid cell resolutions.

### Warming tolerance (WT) and Thermal safety margin (TSM) for the active period

Following Deutsch *et al*.^11^, thermal performance was estimated as the intrinsic rate of population growth (r_max_) across a range of constant temperatures. This value is a direct estimate of Darwinian fitness, and is thus a proper fitness estimate for each species^39^. T_min_, T_opt_ and T_max_ for 38 species at the location of collection was obtained from the data set in Deutsch *et al*.^11^. Thereafter, we estimated warming tolerance (WT) and thermal safety margin (TSM) at the site of collection for each species using the formulas WT = T_max_ − T_hab_, and TSM = T_opt_ − T_hab_ respectively, using the predicted temperature in 2080. As stated above, we calculated the yearly habitat temperature in two ways: using a 12-month approach considering each month mean (T_hab_; same as in Deutsch *et al*.^11^), and our new approach using only means of months where the temperature was >10 °C (T_habA_).

The effects of warming on insect performance was visualized by plotting WT and TSM against latitude of origin for each of the 38 species analyzed by Deutsch *et al*.^11^ for their 2080 warming scenario. We created two different plots: one for the entire year (T_hab_) and one for only the active period (T_habA_). In these plots we also show the predicted temperatures increase at each latitude for the year 2080. To visualize trends in WT and TSM across latitude we used a second-degree polynomial model. These graphs were done in the R package lme4.

To evaluate if tropical species were more sensitive to climate change compared to temperate species we calculated two temperature sensitivity indices (1): WT - predicted mean temperature (ΔT in Fig. 1); and  (2) TSM – predicted mean temperature increase. Again, we did so using both T_hab_ and T_habA_. We define the tropics as the area between 23.5 °N and 23.5 °S^40^. This gave us a total of 6 tropical species and 32 temperate species. The differences in temperature sensitivity indices between tropical and temperate species were tested with t-tests using Minitab version 17.

## Results

As originally reported by Deutsch *et al*.^11^, warming tolerances (WT) and thermal safety margins (TSM) increased steeply with latitude (Fig. 2A,B,) when we calculated these metrics using the annual mean habitat temperature T_hab_ as in Deutsch *et al*.’ s^11^ original analysis. The pattern was significantly better described by a quadratic regression model than a linear model (WT: F_1,35_ = 22.37, P < 0.001; TSM: F_1,35_ = 29.19, P < 0.001). Similar to previous studies, tropical species (below 23° latitude) had on average significantly narrower warming tolerances (t-test, t = 3.62, P = 0.003) and thermal safety margins (t = 2.83, P = 0.015) than temperate species (above 23° latitude). Furthermore, one tropical species and one temperate species was predicted to experience mean annual temperatures that exceeded their thermal safety margin by 2080 (dots below the red line in Fig. 2B). Because the data set is heavily biased towards temperate species, this represent 17% of tropical species and 1% or temperate species being identified as at risk from warming.

**Figure 3:**
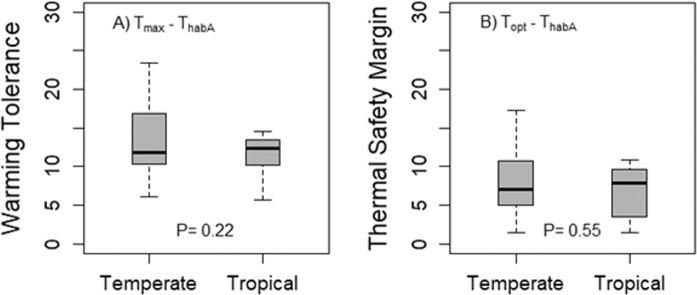
Difference in warming tolerance (**A**) and thermal safety margins (**B**) between temperate and tropical species when accounting for latitudinal differences in insect activity periods. Box plots show median, quartiles and range. P-values are based on t-tests. Tropical areas are defined as regions between 23°N and 23°S latitude.

In contrast, WT and TSM showed a considerably flatter latitudinal trend when we accounted for latitudinal differences in insect activity periods by calculating these metrics using the mean temperature during the active season T_habA_ (Fig. 2C,D,). The pattern was still significantly better described by a quadratic regression model than a linear model (WT: F1,35 = 10.15, P = 0.003; TSM: F1,35 = 11.21, P = 0.002). However, after accounting for activity periods, tropical and temperate species did not differ overall in either warming tolerance (Fig. 3A; t-test, t = 1.30, d.f. = 8.9, P = 0.22) or thermal safety margin (Fig. 3B; t-test, t = 0.63, d.f. = 7.43, P = 0.55) based on currently available data. Moreover, one tropical species and three temperate-zone species were predicted to experience mean temperatures during the active season that exceeded their thermal safety margins by 2080 (Fig. 2D). This analysis accounting for latitudinal differences in insect active periods thus identified 17% of tropical species and 9% of temperate species as at risk to warming.

Accounting for insect activity periods had a greater impact on the predicted warming tolerances and safety margins in temperate than tropical species. Warming tolerances calculated using the mean annual temperature T_hab_ were significantly higher than those calculated using the mean temperature during the active season T_habA_ for temperate (t-test, t = 3.00, d.f. = 53.3, P = 0.004) but not tropical species (t = −0.008, d.f. = 10, P = 0.99). Likewise, thermal safety margins were significantly higher when calculated using T_hab_ than T_habA_ for temperate (t = 3.22, d.f. = 53.3, P = 0.002) but not tropical species (t = −0.007, d.f. = 10, P = 0.99).

## Discussion

Our study suggests that temperate and tropical insects are more similar in their vulnerability to climate warming than expected based on several recent models. These findings support some recent studies finding that ectotherms of temperate and tropical species might differ far less in response to climate change than predicted in past studies^21–25^. Specifically, we find that previous analyses that have compared thermal optima and maxima (T_opt_ and T_max_) with the annual mean habitat temperature (T_hab_) have greatly overestimated the warming tolerances (WT) and thermal safety margins (TSM) of temperate species (Fig. 2A,B), but not those of tropical species. When we account for differences in seasonal activity patterns across latitude, by calculating the mean temperature during the months of the year when insects are developmentally active T_habA_, increases in mean WTs and TSMs with latitude becomes considerably smaller in magnitude than reported in several earlier studies (Fig. 2C,D). This discrepancy simply arises because our metric T_habA_ is not biased downward by including very cold winter temperatures that are not experienced by developmentally active insects at higher latitudes.

Our analyses highlight the striking variability in warming tolerances and thermal safety margins in temperate species (Fig. 2C,D). This variability, together with the weak latitudinal trend in WT and TSM, does not support the generalization that tropical species are more vulnerable overall than temperate species (Fig. 3), although it should be noted that on average, TSM and WT tends to increase non-linearly with latitude here as in previous studies. The weak differences we observe between temperate and tropical taxa contrasts with the results from several earlier studies^4,11,15,17,18,20,41^ that have not accounted for activity periods.

We find the variation in TSM in the temperate zone to be so great that three temperate species – but only one tropical species – showed reduced fitness following the 5 °C increase in average temperatures forecasted by 2080 under IPCC scenario A2 (Fig. 2D; points falling under the red line). This represents 9% and 17% of temperate and tropical species studied, respectively. These relatively similar level of risk stands in stark contrast with the predictions from Deutsch *et al*.’s^11^ original analysis. While their analysis also showed large levels of variation in WT and TSM for temperate species (c.f. Fig. 2B), the mean WT and TSM was estimated to be so high for temperate species that all insects above 40°N were predicted to experience increased fitness even after a 10 °C increase in temperature. Clearly, more data on both tropical and high-latitude species is needed before any firm conclusions can be drawn. Such information might be available now, about a decade later. However, the purpose of our study was simply to show that taking seasonal activity into account will change the predictions along a latitudinal gradient.

The effects of climate change will not only be felt through changes in mean temperature, but also from changes in the variance of temperature experienced over annual and diurnal time scales. In the analyses presented here, we focus on changes in mean temperature for two reasons. Firstly, it enables us to clearly isolate the magnitude of the effect of accounting for insect activity periods across latitude, and secondly, it enables us to directly compare the results from our analyses with those in the original, highly influential, study by Deutsch *et. al*.^11^ However, our findings, that temperate species face a greater risk from warming than commonly expected, reinforce those of two recent studies that have re-analyzed the Deutsch *et al*.^11^ dataset to explore the consequences of increases in both the mean and variance of temperature under climate change^21,30^. Both these studies found that, because mid-latitude environments experience considerably greater variance in temperature than tropical environments, insects from mid-latitudes will experience greater decline in fitness under climate change because they will encounter temperatures above T_opt_ with increasing frequency^21,30^. However, note that this arises because temperate environments show greater variation in in temperature than tropical environments. Our results add to these findings by showing that active insects also experience these temperate environments as warmer than expected based on conditions averaged over the whole year, because temperate insects have shorter active periods. When we add the effect of thermal fluctuations (and increases in thermal variability under warming) to this, we expect that this will further exacerbate the vulnerability of the temperate species that we identify as having narrow thermal safety margins.

The study by Kingsolver *et al*.^21^ is an especially relevant comparison, as they also performed separate analyses using temperature data for both the full year and the ‘growing season’, which they define as May-October in the northern hemisphere (but note that their analysis did not account for differences in the length and timing of the active period across latitude, as we do here). As in our study, Kingsolver *et al*.^21^ found that a considerably greater proportion of mid-latitude species (20–40° absolute latitude) will experience declines in fitness when we only consider the conditions they experience during the growing season. In addition, Kingsolver *et al*.^21^ also found that responses were especially heterogeneous at mid-latitudes, with some species showing increases, and some species decreases, in fitness, which is in concert with our findings. Hence, by using a somewhat different approach we found similar results as Kingsolver *et al*.^21^, and thus our study adds up to the recent suggestions that tropical and temperate species might have similar vulnerability to climate change^21–25^.

Estimates of WT and TSM from TPCs for fitness (*r*) represent a highly valuable source of data on species’ vulnerabilities to warming, as they have been estimated for a relatively large number of species using a consistent methodology, and capture the overall effect of temperature on a set of complex underlying processes that are intrinsically linked to the demographic rates of populations. However, it is becoming increasingly clear that more complex models will be needed to predict the response of individual species with any certainty. Mechanistic models, tailor-made for specific organisms, are becoming increasingly sophisticated. These incorporate, for example, the effects of thermoregulatory behavior^25^, capacity for thermal acclimation^29^, response to the duration and intensity of extreme temperatures^31^, genetic (co)variances^33^ and interspecific interactions^34,42^. However, to apply such mechanistic models on a global scale will require a large amount of data that is currently not available for more than a handful of species. Therefore, we believe that TPCs for fitness will continue to serve an important role for identifying the broad trends in the vulnerability to warming. Going forward, we strongly urge researchers to consider latitudinal differences in active periods when exploring this rich source of information.

Our analysis ignores sources of selection that occurs during the non-active period. This decision may be defensible when analyzing changes in the expression of performance traits (feeding, growth, locomotion, etc) that are only expressed during this part of the year. However, because fitness represents the joint outcome of survival and reproductive success, it may be especially important to also account for mortality that occur during the non-active periods. Likewise, because TPCs for fitness are typically estimated under controlled laboratory conditions, it is important to note that published fitness estimates also ignore many other important sources of selection, including predation, competition, resource availability, etc, that occur both within and outside the active period. We also note that the information currently available on insect thermal responses is highly incomplete and biased. Large areas remain entirely unsampled (Supporting information, Fig. S4), most notably all of South America, and very few populations above 50°N have been studied (none above 53°N). Hence, for a good understanding on how climate change will affect insect species above 50°N much more data on fitness estimates at different temperatures are urgently needed.

In summary, our work reveals that temperate species have considerably narrower safety margin to warming than suggested by several earlier analysis. Because temperate ectotherm species also harbor considerable variation in T_opt_ and T_max_ (Supporting information, Table S2) it will be important to have much more data on thermal tolerance and fitness components across latitudes before we can make more precise predictions on the impacts of climate change.

## Supplementary information

Supplementary Information

Supplementary Table S2
