# Supplementary material for: Temperate insects with narrow seasonal activity periods can be as vulnerable to climate change as tropical insect species
Source: Sci Rep. 2020 Jun 1;10:8822. doi: 10.1038/s41598-020-65608-7 (PMC7264184; doi:10.1038/s41598-020-65608-7)
Supplement: Supplementary file 1 — Supplementary Information [file 41598_2020_65608_MOESM1_ESM.docx]

Supplementary information

**Temperate insects with narrow seasonal activity periods can be as vulnerable to climate change as tropical insect species**

Frank Johansson^1,5^, Germán Orizaola^1,2,3^ and Viktor Nilsson-Örtman^4^

^1^ Uppsala University, Animal Ecology, Department of Ecology and Genetics, Evolutionary Biology Center, Norbyvägen 18D, S-75236 Uppsala, Sweden.

^2^ UIB-Research Unit of Biodiversity (Univ. Oviedo-CSIC-Princ. Asturias), c/ Gonzalo Gutiérrez Quirós s/n, 33600 Mieres-Asturias, Spain.

^3^ University of Oviedo, Zoology Unit, Dept Organisms & Systems, c/Rodrigo Uría s/n, 33071 Oviedo-Asturias, Spain.

^4^ Lund University, Department of Biology, Evolutionary Ecology Unit, Sölvegatan 12, S-22362 Lund, Sweden.

^5^ [frank.johansson@ebc.uu.se](mailto:frank.johansson@ebc.uu.se)

Supporting information, Table S1.

T_dmin_ for the insects used to calculate average T_dmin_ and regression of T_dmin_ against latitude. Note that more species are available in Dixon et al. (2007: Functional Ecology), but we were not able to retrieve the origin of latitude for those species.

Supporting information, Table S3.

Geographical position, systematic position, and ecological characteristics of the 38 species for which TPCs for fitness have been estimated. For a list of full references see Deutsch et al. (2008).


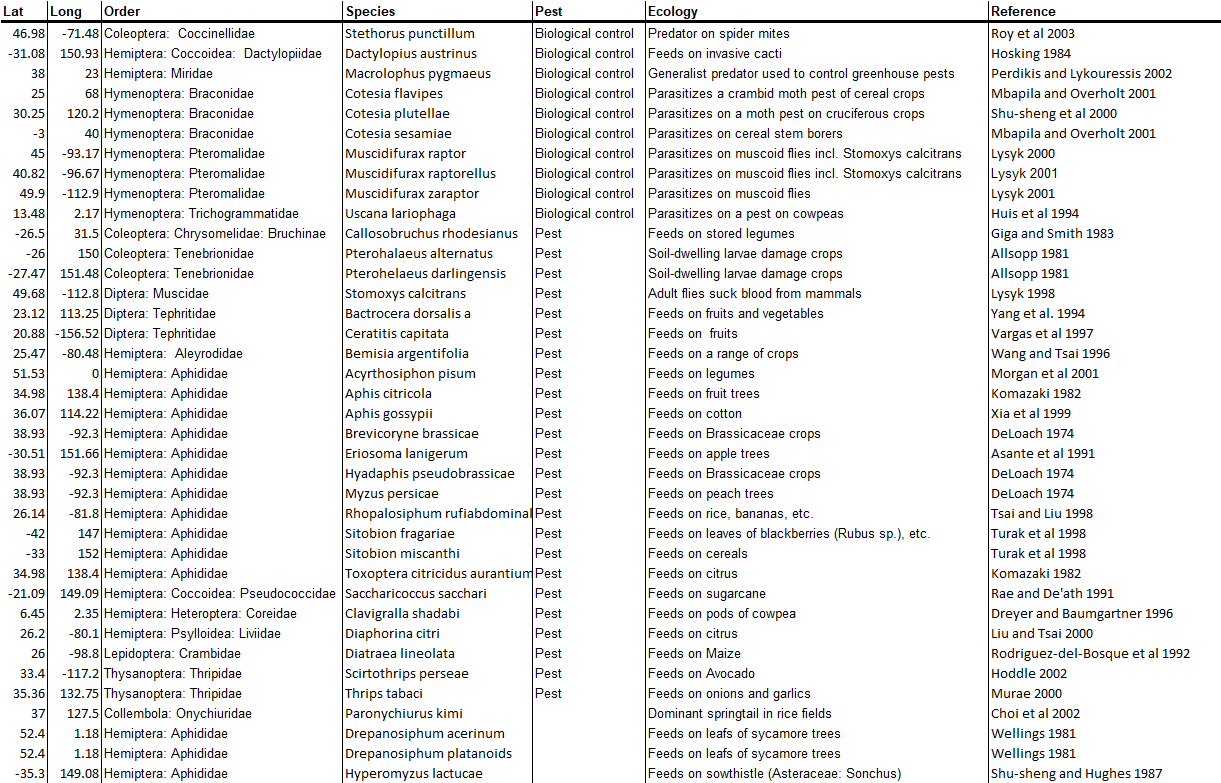


Supporting information Figure S4


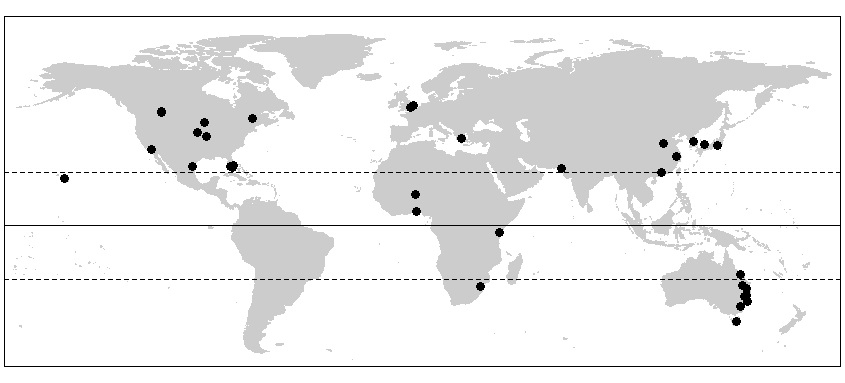


Figure 4. Location of origin of the 38 insect species for which TPCs for fitness have been estimated.
